# Comparison of depressive symptoms and inflammation between sexual minorities and heterosexuals using NHANES study of 8538 participants

**DOI:** 10.1038/s41598-022-07702-6

**Published:** 2022-03-08

**Authors:** Amandeep Mann, Ava Chan, Atharva Rohatgi, Michelle Ann Caesar, Juno Obedin-Maliver, Daniel S. Kapp

**Affiliations:** 1 Palo Alto Medical Foundation Research Institute, 795 El Camino Real, Palo Alto, CA 94301 USA; 2 California Pacific Medical Center Research Institute, 475 Brannan St, San Francisco, CA 94107 USA; 3 Department of Obstetrics and Gynecology, Stanford University School of Medicine, 900 Blake Wilbur Dr. Ste W0050, Stanford, CA 94304 USA; 4 Department of Radiation Oncology, Stanford University School of Medicine, 875 Blake Wilbur Dr, Stanford, CA 94304 USA

**Keywords:** Biomarkers, Health care, Depression

## Abstract

The present study aims to compare the rate of depressive symptoms and inflammation levels between sexual minorities and heterosexuals. Data were obtained from the National Health and Nutrition Examination Survey from 2005 to 2010. Depressive-related symptoms were measured using the Patient Health Questionnaire-9 scoring system. C-reactive protein was analyzed with the Behring Nephelometer. Of 8538 participants, 95.8% self-reported as heterosexual and 4.2% as sexual minority. Depressive symptoms were reported in 7.1% of heterosexuals compared to 15.8% in sexual minorities (*P* = 0.001). In heterosexuals, C-reactive protein was higher in those with depressive symptoms compared to those without (*P* < 0.001). In sexual minorities, similar results were found, however, it was statistically insignificant. The intersection group of black sexual minority females reported the highest rate of depressive symptoms at 33.4%. We found that depressive symptoms were higher in sexual minorities compared to heterosexuals. Furthermore, systemic inflammation was highest in the intersection group of black sexual minority females.

## Introduction

According to the Centers for Disease Control and Prevention, nearly one-fifth of adults reported depressive symptoms annually^1^. Compared to heterosexuals, sexual minorities have higher rates of depression and more likely to manifest symptoms of anxiety possibly related to greater social stigma regarding their sexuality^2,3^. Additionally, due to biological and external factors, the rates of depression vary based on sex, race, and socioeconomic status^4,5^. Previous studies indicate that depression is more prevalent among women, racial minorities, and those of lower socioeconomic status^5–7^. However, the intersection of sexual minority groups with respect to these demographic factors has not been thoroughly analyzed. Additionally, demographic intersections have been previously found to impact mental health outcomes. Furthermore, there may be a proportion of patients with depression that can be explained through chronic inflammation^8^. Previous studies have cited systemic inflammation (measured by CRP levels) due to social stigma and a lack of safety as a possible indicator for poor health outcomes among sexual minorities^9^. Although systemic inflammation has been shown to be associated with depressive symptoms, this relationship is not clearly defined after adjusting for sex and racial groups^4,5^. In this project, we compared the rates of depression and inflammation levels with respect to sexual orientation. In addition, we also examined the intersection of demographic and clinical factors with respect to depression rates.

## Methods

### Data source

Data was collected from the National Health and Nutrition Examination Survey (NHANES) which is publicly available. NHANES is a national survey conducted by the Centers for Disease Control and Prevention that utilizes a combination of in-home interviews and physical examinations to evaluate the health and nutritional status of U.S. residents^10^. Depressive symptoms and C-reactive protein (CRP) data were available between 2005 and 2010, and then supplemented with data on demographics, healthcare characteristics, and sexual orientation.

#### Inclusion/exclusion criteria

We elected to include participants aged 20–59 (*N* = 9404) years since this group responded to sexual behavior and smoking behavior questionnaires. From this we excluded female participants who were pregnant (431). Individuals with missing information on depressive symptoms (20), marital status (7), education (7), body mass index (45) demographic, socioeconomic characteristics, CRP levels (349), self-identified sexual orientation, and depressive-related symptoms (20) were excluded from our analysis. Since prior studies have shown that abnormally high CRP levels have been associated with active infection instead of chronic inflammation, we also excluded those with CRP ≥ 10 mg/dL (7) from our analytic dataset. After incorporating the exclusion criteria our final analytic dataset came out to be 8,538.

#### Outcomes and measures

Individuals were defined collectively as sexual minority if they self-reported their sexual orientation as either gay, lesbian, or bisexual. Depressive-related symptoms were measured based on the nine-item Patient Health Questionnaire (PHQ-9) using a scoring system ranging from 0 (not at all) to 3 (nearly every day). Participants with a score ≥ 10 indicate depressive symptoms^8^. CRP, a serum inflammatory biomarker, was analyzed with the Behring Nephelometer using anti-CRP antibody latex particles. Participants’ age was dichotomized using the median value, 40 years. Race was categorized as non-Hispanic white, non-Hispanic black, Hispanic, and another race (includes Asian race, mixed race, and other race). Individual’s level of education in our cohort was grouped as less than high school, high school, and above high school. To define income levels, we used the poverty-income ratio (PIR) which is the ratio of family income to poverty threshold. Using this ratio and the U.S Census definition of income categories, we categorized income levels as poverty (PIR < 1), low income (1.0 ≤ PIR < 2.0), middle income (2.0 ≤ PIR < 4.0), and high income (PIR ≥ 4.0). We elected to combine “poverty” and “low income” groups for our analysis. In our analysis marital status was grouped as “single”, “married or with partner”, “divorced or separated or widowed.” Participants' smoking status was either reported as a current, former, or non-smoker. Based on CDC on defining body mass index (BMI) categories, we categorized this variable as underweight (< 18.5 kg/m^2^), normal (18.5–24.9 kg/m^2^), overweight (25.0–29.9 kg/m^2^), and obese (≥ 30.0 kg/m^2^).

#### Data analysis

Using SAS Enterprise Guide Version 7.1 (SAS Institute Inc., Cary, NC, USA), chi-square, t-tests, and multivariate logistic regression were employed for statistical methods. Incorporating cluster, strata, and weighted variables in our analysis accounted for sampling weights and complexity of survey design and ensured that the oversampling of any groups did not occur. Covariates for the multivariable logistic regression model were determined by conducting bi-variable chi-square tests. Variables found to be significantly associated with depressive symptoms were included as a covariate. Associations were considered to be statistically significant if the two-sided *P*-value is 0.05 or less. NHANES data is publicly available and the datasets used in this study can be found online.

### Consent to participate/Consent to publish

This study was deemed exempt from IRB approval as it contains de-identified information.

### Human and animal rights

All statistical methods for this NHANES study were conducted in accordance with the Declaration of Helsinki.

## Results

**Figure 1:**
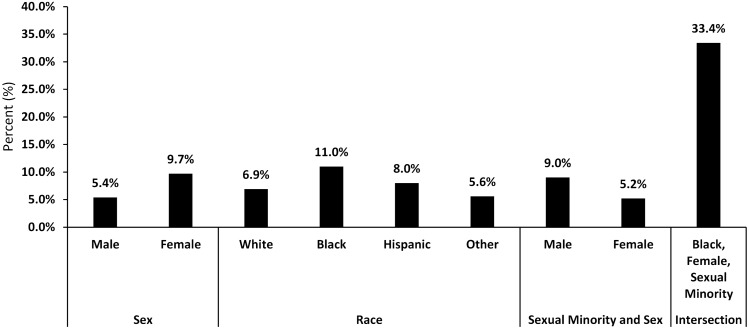
Depressive symptoms by sex, race, and sexual minority.

Of 8538 participants (median age: 40 years, range: 20–59), heterosexuals comprised 95.8% and 4.2% were sexual minorities. Depressive symptoms were reported in 5.4% of males versus 9.7% of females (*P* < 0.001) and 7.1% of the heterosexuals versus 15.8% of sexual minorities (*P* = 0.001) (Table 1). From 2005 to 2010, the sexual minorities with depressive symptoms have increased from 12.7 to 22.3% (*P* = 0.16) whereas heterosexuals remained stable over time (5.5–7.7%). C-reactive protein was significantly higher in those with depressive symptoms compared to those without depressive symptoms in heterosexuals (0.54 mg/dL vs. 0.35 mg/dL; *P* < 0.001) and sexual minorities (0.55 mg/dL vs. 0.35 mg/dL; *P* = 0.13), though statistically insignificant (Table 2). In an intersection analysis of females who are black and sexual minorities, their depressive symptoms were reported at 33.4% compared to only 5% of white heterosexual males (*P* = 0.001) (Fig. 1), with corresponding CRP levels of 0.52 mg/dL and 0.43 mg/dL (*P* = 0.01) (Table 3). On multivariate analysis, sexual minorities (OR = 2.05, 95% CI: 1.38–3.04; *P* = 0.001), females (OR = 1.81, 95% CI: 1.51–2.16; *P* < 0.001), and those with elevated CRP levels (OR = 1.22, 95% CI: 1.09–1.40; *P* = 0.001) were independently associated with depressive symptoms (Table 4).

**Table 1:** Demographics, socioeconomic status, and behavioral characteristics by depressive symptoms.

Factors	Overall (*N* = 8538)	Depressive symptoms^a^ (%)	No depressive symptoms (%)	*P*-value
**Sexual orientation**				0.001^b^
Heterosexuals	4.2%	7.1%	92.9%	
Sexual minority	95.8%	15.8%	84.2%	
**Age (years)**				0.01^b^
Mean (Standard deviation)	40 (0.19)	41 (0.40)	40 (0.19)	
Median (Range)	40 (20–59)	43 (20–59)	40 (20–59)	
≤ 40 years	50.1%	6.6%	93.4%	
> 40 years	49.9%	8.3%	91.7%	
**Sex**				< 0.001^b^
Male	51.3%	5.4%	94.6%	
Female	48.7%	9.7%	90.3%	
**Race**				
White	69.4%	6.9%	93.1%	< 0.001^b^
Black	11.1%	11.0%	89.0%	
Hispanic	13.8%	8.0%	92.0%	
Other^c^	5.7%	5.6%	94.4%	
**Education**				< 0.001^b^
Below high school	15.6%	12.4%	87.6%	
High school	23.2%	8.6%	91.4%	
AA and some college	32.8%	8.1%	91.9%	
BA and above	28.4%	3.2%	96.8%	
**Income**				< 0.001^b^
Low	22.6%	14.2%	85.8%	
Middle	31.5%	7.6%	92.4%	
High	45.9%	4.1%	95.9%	
**Marital status**				< 0.001^b^
Single	20.4%	7.6%	92.4%	
Married or with partner	65.6%	5.9%	94.1%	
Divorced, widowed, or separated	14.0%	14.7%	85.3%	
**Smoking status**				< 0.001^b^
Non-smokers	53.9%	5.3%	94.7%	
Former	20.0%	6.1%	93.9%	
Current	26.1%	13.0%	87.0%	
**BMI category**				< 0.001^b^
Underweight	2.2%	7.1%	92.9%	
Normal weight	31.1%	6.4%	93.6%	
Overweight	32.3%	6.3%	93.7%	
Obese	34.4%	9.5%	90.5%	
**CRP (mg/dL)**				
Mean (Standard deviation)	0.16 (0.01)	0.54 (0.04)	0.35 (0.00)	< 0.001^d^
Median (Range)	0.16 (0.01–9.16)	0.26 (0.01–8.1)	0.15 (0.01–9.16)	

^a^Participants with PHQ-9 score ≥ 10 were categorized as having depressive symptoms.

^b^Chi-square test was used to calculate the *P*-value.

^c^Other race includes Asians, Multi-race, and other race not specifically identified in NHANES.

^d^T-test was used to calculate the *P*-value.

**Table 2:** Demographics, socioeconomic status, and behavioral characteristics by depressive symptoms and sexual orientation.

Factors	Heterosexual (*N* = 8172)			Sexual minority (*N* = 366)		
	Depressive symptoms^a^ (%)	No depressive symptoms (%)	*P*-value	Depressive symptoms^a^ (%)	No depressive symptoms (%)	*P*-value
**Age**			0.002^b^			0.41^b^
≤ 40 years	6.0	94.0		17.0	83.0	
> 40 years	8.2	91.8		13.8	86.2	
**Sex**			< 0.001^b^			0.002^b^
Male	5.2	94.8		9.0	91.0	
Female	9.1	90.9		20.7	79.3	
**Race**			0.001^b^			0.43^b^
White	6.6	93.4		14.4	85.6	
Black	10.3	89.7		23.4	76.6	
Hispanic	7.8	92.2		14.8	85.2	
Other^c^	5.3	94.7		16.0	84.0	
**Education**			< 0.001^b^			0.04^b^
Below high school	11.8	88.2		25.8	74.2	
High school	8.2	91.8		20.4	79.6	
AA and some college	7.7	92.3		16.7	83.3	
BA and above	2.9	97.1		7.8	92.2	
**Income**			< 0.001^b^			< 0.001^b^
Low	13.4	86.6		28.9	71.1	
Middle	7.1	92.9		16.7	83.3	
High	4.0	96.0		5.5	94.5	
**Marital status**			< 0.001^b^			0.96^b^
Single	6.8	93.2		15.2	84.8	
Married or with partner	5.6	94.4		16.0	84.0	
Divorced, widowed, or separated	14.6	85.4		17.1	82.9	
**Smoking status**			< 0.001^b^			0.003^b^
Non-smokers	5.1	94.9		11.4	88.6	
Former	6.0	94.0		8.2	91.8	
Current	12.3	87.7		24.3	75.7	
**BMI category**			0.001^b^			0.13^b^
Underweight	7.3	92.7		3.1	96.9	
Normal weight	6.1	93.9		13.0	87.0	
Overweight	6.1	93.9		12.8	87.2	
Obese	8.9	91.1		20.8	79.2	
**Mean CRP (mg/dL)**	0.54	0.35	< 0.001^d^	0.55	0.35	0.13^d^

^a^Participants with PHQ-9 score ≥ 10 were categorized as having depressive symptoms.

^b^Chi-square test was used to calculate the *P*-value.

^c^Other race includes Asians, Multi-race, and other race not specifically identified in NHANES.

^d^T-test was used to calculate the *P*-value.

**Table 3:** Demographics, socioeconomic status, and behavioral characteristics by depressive symptoms and sexual orientation stratified by sex.

Factors	Male						Female					
	Heterosexual			Sexual minority			Heterosexual			Sexual minority		
	Depressive symptoms^a^ (%)	No depressive symptoms (%)	*P*-value	Depressive symptoms^a^ (%)	No depressive symptoms (%)	*P*-value	Depressive symptoms^a^ (%)	No depressive symptoms (%)	*P*-value	Depressive symptoms^a^ (%)	No depressive symptoms (%)	*P*-value
**Age**			0.04^b^			0.93^b^			0.003^b^			0.84^b^
≤ 40 years	4.3%	95.7%		9.2%	90.8%		8.0%	92.0%		21.1%	78.9%	
> 40 years	6.2%	93.8%		8.9%	91.1%		10.1%	89.9%		19.7%	80.3%	
**Race**			0.16^b^			0.80^b^			< 0.001^b^			0.24^b^
White	5.0%	95.0%		8.6%	91.4%		8.3%	91.7%		18.3%	81.7%	
Black	7.7%	92.3%		7.8%	92.2%		12.7%	87.3%		33.4%	66.6%	
Hispanic	4.8%	95.2%		13.4%	86.6%		11.7%	88.3%		16.5%	83.5%	
Other^c^	5.2%	94.8%		7.4%	92.6%		5.4%	94.6%		27.3%	72.7%	
**Education**			< 0.001^b^			0.004^b^			< 0.001^b^			0.72^b^
Below high school	7.9%	92.1%		28.0%	72.0%		17.0%	83.0%		24.9%	75.1%	
High school	6.2%	93.8%		12.4%	87.6%		10.7%	89.3%		23.5%	76.5%	
AA and some college	5.5%	94.5%		10.9%	89.1%		9.8%	90.2%		20.4%	79.6%	
BA and above	2.3%	97.7%		2.7%	97.3%		3.5%	96.5%		15.2%	84.8%	
**Income**			< 0.001^b^			< 0.001^b^			< 0.001^b^			0.01^b^
Low	9.6%	90.4%		23.5%	76.5%		17.3%	82.7%		31.6%	68.4%	
Middle	5.5%	94.5%		11.9%	88.1%		9.0%	91.0%		19.5%	80.5%	
High	3.0%	97.0%		0.8%	99.2%		5.2%	94.8%		10.8%	89.2%	
**Marital status**			< 0.001^b^			< 0.001^b^			< 0.001^b^			0.51^b^
Single	5.9%	94.1%		14.4%	85.6%		7.8%	92.2%		16.0%	84.0%	
Married or with partner	4.0%	96.0%		2.2%	97.8%		7.5%	92.5%		24.0%	76.0%	
Divorced, widowed, or separated	11.5%	88.5%		4.5%	95.5%		16.9%	83.1%		23.1%	76.9%	
**Smoking status**			< 0.001^b^			< 0.001^b^			< 0.001^b^			0.02^b^
Non-smokers	3.3%	96.7%		7.5%	92.5%		6.6%	93.4%		15.1%	84.9%	
Former	4.9%	95.1%		0.0%	100.0%		7.4%	92.6%		12.4%	87.6%	
Current	8.9%	91.1%		14.9%	85.1%		17.0%	83.0%		30.1%	69.9%	
**BMI category**			0.04^b^			0.26^b^			< 0.001^b^			< 0.001^b^
Underweight	1.4%	98.6%		16.9%	83.1%		10.8%	89.2%		0.0%	100.0%	
Normal weight	6.4%	93.6%		6.6%	93.4%		6.0%	94.0%		17.8%	82.2%	
Overweight	4.1%	95.9%		6.5%	93.5%		9.2%	90.8%		20.8%	79.2%	
Obese	5.8%	94.2%		13.6%	86.4%		12.2%	87.8%		24.7%	75.3%	
**Mean CRP (mg/dL)**	0.50	0.29	0.005^d^	0.19	0.24	0.38^d^	0.60	0.41	0.001^d^	0.66	0.44	0.19^d^

^a^Participants with PHQ-9 score ≥ 10 were categorized as having depressive symptoms.

^b^Chi-square test was used to calculate the *P*-value.

^c^Other race includes Asians, Multi-race, and other race not specifically identified in NHANES.

^d^T-test was used to calculate the *P*-value.

**Table 4:** Adjusted odds ratio of depressive symptoms between 2005 and 2010.

Factors	Odds ratio^a^	95% Confidence interval	*P*-value
**Sexual orientation**			
Heterosexuals	1		
Sexual minority	2.05	1.38–3.04	0.001
**Age**			
≤ 40 years	1		
> 40 years	1.39	1.14–1.70	0.002
**Sex**			
Male	1		
Female	1.81	1.51–2.16	< 0.001
**Race**			
White	1		
Black	1.14	0.92–1.40	0.23
Hispanic	0.90	0.70–1.15	0.38
Other^b^	0.90	0.53–1.53	0.68
**Education**			
B.A or above	1		
Below high school	2.27	1.51–3.40	< 0.001
High school	1.81	1.26–2.60	0.002
A.A or some college	1.88	1.33–2.66	0.001
**Income**			
High	1		
Middle	1.49	1.15–1.94	0.004
Low	2.59	2.15–3.13	< 0.001
**Marital status**			
Married or with partner	1		
Single	1.13	0.90–1.42	0.29
Divorced, widowed, or separated	1.91	1.55–2.33	< 0.001
**Smoke**			
Non-smokers	1		
Former	1.11	0.81–1.52	0.52
Current	2.05	1.65–2.56	< 0.001
**BMI category**			
Underweight	1		
Normal weight	1.37	0.67–2.80	0.38
Overweight	1.43	0.69–2.97	0.33
Obese	1.86	0.91–3.81	0.09
**C-reactive protein (mg/dL)**	1.22	1.09–1.40	0.001

^a^Odds ratio of depressive symptoms.

^b^Other race includes Asians, Multi-race, and other race not specifically identified in NHANES.

## Discussion

Sexual minorities reported more depressive symptoms compared to heterosexuals. Of note, nearly one-third of the female Black sexual minority intersection group had depressive symptoms. Furthermore, sexual minorities with depressive symptoms had significantly higher inflammation expressed by C-reactive protein.

Our results found that sexual minority individuals had more depressive symptoms. Others have also found that depression was significantly higher in LGBT individuals compared to heterosexuals^11^. This finding supports the minority stress theory, as described by Brooks, which postulates that exposure to discrimination, stigma, and prejudice on the basis of sexual orientation and gender identity negatively impacts sexual minorities^12^. LGBT individuals may have internalized feelings of homophobia or expectations of rejection that may contribute to their depressive symptoms^3^.

A previous study showed that Black and Latino same-sex-attracted individuals reported higher rates of depressive symptoms compared to their heterosexual counterparts^13^. However, this study did not evaluate the intersection of sex, race, and sexual orientation with respects to depression. Our data showed that black female sexual minorities had the highest rates of depressive symptoms. To our knowledge, this is one of the few studies that have evaluated the intersection between sex, race, and sexual orientation.

Our study identified that the intersectional group of black, sexual minority, and female sex had the highest level of C-reactive protein compared to other groups. The intersection of social categorizations including sex, race, and sexual orientation result in overlapping, interdependent systems of advantages or disadvantages. In this report, we showed that black female sexual minorities experience overlapping prejudices that may further increase stress levels^14^. On the other hand, other investigators have found that sexual minority men had higher levels of C-reactive protein compared to sexual minority women^15^. Clearly, the role of inflammation warrants further analysis given the multiple biologic and external factors that may confound these results.

Such factors such as sedentary lifestyle, diet, and disordered sleep were not included in our study. Medical illnesses including cancer, autoimmune, inflammatory disorders, and cardiovascular disease are also limited. Additionally, there is much unknown in regard to childhood impact, with factors such as childhood maltreatment and emotional as well as physical trauma.

## Conclusion

Female black sexual minorities reported significantly higher depressive symptoms compared to their white heterosexual counterparts. Based on our findings, further studies are warranted to address the reasons behind the disparities in the incidences and treatment of depression in correlation with systemic inflammation biomarkers. Additionally, this study highlights the need for improved social support systems and clinical mental health treatment for these at-risk subgroups.

## Data Availability

The datasets used during the current study are available from the National Health and Nutrition Examination Survey (NHANES). The data can be accessed from the website: https://www.cdc.gov/nchs/nhanes/index.htm

## References

[1] Villarroel MA, Terlizzi EP (2020). Symptoms of Depression Among Adults: United States, 2019. NCHS Data Brief, no 379.

[2] Borgogna NC, McDermott RC, Aita SL, Kridel MM (2019). Anxiety and depression across gender and sexual minorities: Implications for transgender, gender nonconforming, pansexual, demisexual, asexual, queer, and questioning individuals. Psychol. Sex. Orientat. Gend. Divers..

[3] Krueger EA, Meyer IH, Upchurch DM (2018). Sexual orientation group differences in perceived stress and depressive symptoms among young adults in the United States. LGBT Health.

[4] Beydoun MA, Obhi HK, Weiss J, Canas JA, Beydoun HA, Evans MK, Zonderman AB (2020). Systemic inflammation is associated with depressive symptoms differentially by sex and race: A longitudinal study of urban adults. Mol. Psychiatr..

[5] Altemus M, Sarvaiya N, Neill EC (2014). Sex differences in anxiety and depression clinical perspectives. Front. Neuroendocrinol..

[6] Hooker K, Phibbs S, Irvin VL (2019). Depression among older adults in the united states by disaggregated race and ethnicity. Gerontologist.

[7] Assari S (2019). Race, depression, and financial distress in a nationally representative sample of American adults. Brain Sci..

[8] Miller AH, Raison CL (2016). The role of inflammation in depression: From evolutionary imperative to modern treatment target. Nat. Rev. Immunol..

[9] Diamond LM, Dehlin AJ, Alley J (2021). Systemic inflammation as a driver of health disparities among sexually-diverse and gender-diverse individuals. Psychoneuroendocrinology.

[10] About the national health and nutrition examination survey. (2017). Retrieved from https://www.cdc.gov/nchs/nhanes/about_nhanes.htm.

[11] Jha MK, Minhajuddin A, Gadad BS (2017). Can C-reactive protein inform antidepressant medication selection in depressed outpatients? Findings from the CO-MED trial. Psychoneuroendocrinology.

[12] Holman EG (2018). Theoretical extensions of minority stress theory for sexual minority individuals in the workplace: A cross-contextual understanding of minority stress processes. J. Fam. Theory Rev..

[13] Russell ST, Fish JN (2016). Mental health in lesbian, gay, bisexual, and transgender (LGBT) youth. Annu. Rev. Clin. Psychol..

[14] Doyle DM, Molix L (2016). Minority stress and inflammatory mediators: Covering moderates associations between perceived discrimination and salivary interleukin-6 in gay men. J. Behav. Med..

[15] Everett BG, Rosario M, McLaughlin KA, Austin SB (2014). Sexual orientation and gender differences in markers of inflammation and immune functioning. Ann. Behav. Med..

